# Recent Progress in Porphyrin/g-C_3_N_4_ Composite Photocatalysts for Solar Energy Utilization and Conversion

**DOI:** 10.3390/molecules28114283

**Published:** 2023-05-23

**Authors:** Sudi Chen, Jiajia Wei, Xitong Ren, Keke Song, Jiajie Sun, Feng Bai, Shufang Tian

**Affiliations:** 1 Key Laboratory for Special Functional Materials of Ministry of Education, National and Local Joint Engineering Research Center for High-Efficiency Display and Lighting Technology, School of Materials Science and Engineering, and Collaborative, Innovation Center of Nano Functional Materials and Applications, Henan University, Kaifeng 475004, China; 2 Henan International Joint Laboratory of Medicinal Plants Utilization, College of Chemistry and Molecular Science, Henan University, Kaifeng 475004, China; 3 School of Physics and Electronics, Henan University, Kaifeng 475004, China

**Keywords:** porphyrin, g-C_3_N_4_, solar energy, hydrogen generation, CO_2_ reduction, pollutants degradation

## Abstract

Transforming solar energy into chemical bonds is a promising and viable way to store solar energy. Porphyrins are natural light-capturing antennas, and graphitic carbon nitride (g-C_3_N_4_) is an effective, artificially synthesized organic semiconductor. Their excellent complementarity has led to a growing number of research papers on porphyrin/g-C_3_N_4_ hybrids for solar energy utilization. This review highlights the recent progress in porphyrin/g-C_3_N_4_ composites, including: (1) porphyrin molecules/g-C_3_N_4_ composite photocatalysts connected via noncovalent or covalent interactions, and (2) porphyrin-based nanomaterials/g-C_3_N_4_ composite photocatalysts, such as porphyrin-based MOF/g-C_3_N_4_, porphyrin-based COF/g-C_3_N_4_, and porphyrin-based assembly/g-C_3_N_4_ heterojunction nanostructures. Additionally, the review discusses the versatile applications of these composites, including artificial photosynthesis for hydrogen evolution, CO_2_ reduction, and pollutant degradation. Lastly, critical summaries and perspectives on the challenges and future directions in this field are also provided.

## 1. Introduction

In recent decades, global energy consumption and demand have grown rapidly alongside population and economic growth. However, the traditional carbon-based fossil energy resources, particularly coal, are facing not only a shortage of reserves but also leading to excessive CO_2_ emissions and accumulation in the atmosphere, posing a threat to the environment and ecology. Therefore, developing clean and sustainable new carbon-neutral energy sources has become a pressing scientific challenge. Solar energy is one of the most secure and valuable resources and the largest mineable among all renewable energy sources [1,2,3]. However, the challenge lies in exploring and storing this abundant resource. One promising approach is to convert solar energy into chemical bonds, such as generating hydrogen and oxygen through water splitting or obtaining reduced fuels like methane, methanol, carbon monoxide, or other hydrocarbon species through CO_2_ reduction [4,5,6]. Additionally, solar energy can be utilized to degrade organic pollutants, thus relieving the pressure of environmental pollution [7,8]. The crux of addressing solar energy utilization and conversion lies in developing photocatalysts with higher artificial photosynthesis or photodegradation efficiency.

**Figure 1:**
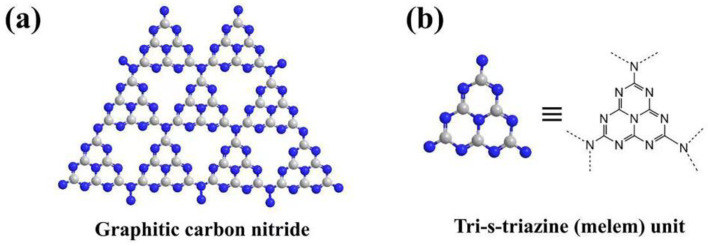
Chemical structure of g-C_3_N_4_ (**a**) and tri-s-triazine (heptazine) (**b**).

The organic semiconductor graphitic carbon nitride (g-C_3_N_4_), depicted in Figure 1a, featuring heptazine rings (Figure 1b) as fundamental structural units, has undergone extensive research and application in the field of photocatalysis over the past decades [9,10,11]. This material offers several advantages, which are outlined as: (1) It is non-toxic, low-cost, and easily prepared with a wide range of nitrogen-rich materials as precursors, including melamine, urea, cyanamide, dicyanamide, and thiourea. (2) It possesses suitable bandgap (about 2.7 eV) and responses to visible-light range. The conduction band (CB) and the valence band (VB) of pristine g-C_3_N_4_ are commonly located at −1.3 and 1.4 eV, respectively, which sufficiently satisfies the requirements of hydrogen and oxygen generation by splitting water, CO_2_ photocatalytic reduction, and organic pollutants degradation from the thermodynamic aspect. Furthermore, in contrast to inorganic semiconductors, the narrow bandgap of g-C_3_N_4_ enables it to harvest the sunlight with a wavelength up to 460 nm, which enables it to utilize solar energy more efficiently. (3) Its two-dimensional (2D) lamellar structure with van der Waals between interlayers not only contributes to adsorbing more reactants and exposing more catalytic active sites, but also facilitates post modification, such as exfoliation, to further increase its specific surface area. (4) It is gifted with distinctive surface properties that facilitates hydrogen bonds or to covalently connect with organic molecules for its surface functional groups, such as amines; it tends to form π-π stacking with other aromatic molecules due to its conjugated structure; it opts to chelate with metal ion for its electron-rich characters, and so on. (5) Thanks to its unique molecular structure, it is not only provided with excellent thermal and chemical stability even under strong acids and alkali conditions, but can also be modified by means of various organic chemistry methods apart from the strategies generally employed for inorganic semiconductors. Therefore, g-C_3_N_4_ has wide application prospects in the field of photocatalysis.

It is unfortunate that g-C_3_N_4_ still faces limitations such as a limited visible light response range, a small specific surface area, and rapid recombination of photo-generated charge carriers. Consequently, numerous strategies have been employed to enhance the photocatalytic performance of g-C_3_N_4_. These approaches encompass various techniques such as nanostructure design, exfoliation, doping with elements and molecules, and the construction of heterojunctions, such as porphyrin/g-C_3_N_4_ heterojunctions [12,13].

**Figure 2:**
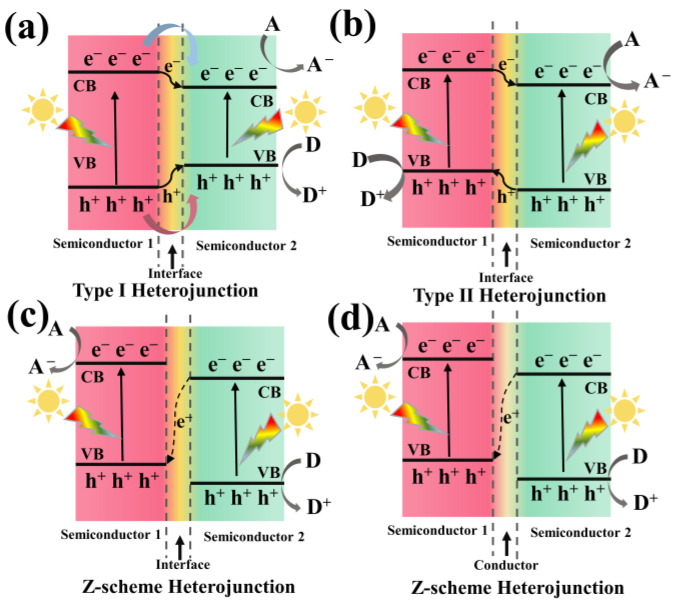
Schematic illustration of the three types of heterojunctions in a semiconductor hybrid: Type I heterojunction (**a**), Type II heterojunction (**b**), and Z-scheme heterojunction (**c**,**d**).

Before delving into porphyrin and porphyrin/g-C_3_N_4_ heterojunctions, it is essential to grasp the concept of the three distinct types of heterojunctions [10,14,15]. In the case of type I band alignment (Figure 2a), semiconductor 1 exhibits a more negative CB and a more positive VB compared to semiconductor 2. When the irradiation light energy equals or exceeds the bandgap of semiconductor 1, the photo-generated electrons or holes tend to migrate to the adjacent CB or VB of semiconductor 2, where charge carriers accumulate. This not only fails to improve the separation efficiency of charge carriers but also diminishes the redox ability. However, in type II band alignment (Figure 2b), the CB and VB potentials are intertwined between semiconductor 1 and semiconductor 2. Photo-induced electrons and holes migrate in opposite directions, creating an internal electric field that enhances the spatial separation of electron-hole pairs and significantly inhibits recombination. Consequently, the oxidation and reduction reaction occur in two semiconductors, but with the diminished redox capacity as a cost. To address this predicament, a Z-scheme heterojunction has been developed. One type is the semiconductor–semiconductor Z-scheme heterojunction (Figure 2c), where photo-induced electrons from semiconductor 2 interact with the positive edge of semiconductor 1, and both the electrons and holes remain in the CB of semiconductor 1 and the VB of semiconductor 2. The other type is the semiconductor–conductor–semiconductor Z-scheme heterojunction (Figure 2d), where an electron conductor such as a noble metal or graphene bridges the two semiconductors, facilitating electron transmission from semiconductor 2 to semiconductor 1. Moreover, this heterojunction maintains the high redox ability.

**Figure 3:**
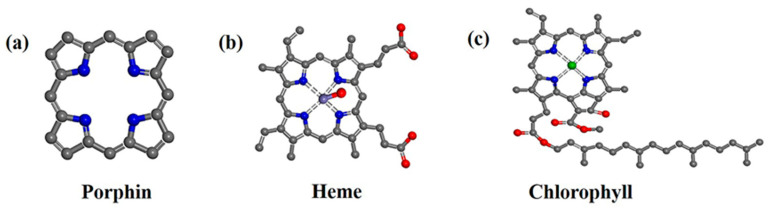
Chemical structure of porphin (**a**), heme (**b**), and chlorophyll (**c**).

**Figure 4:**
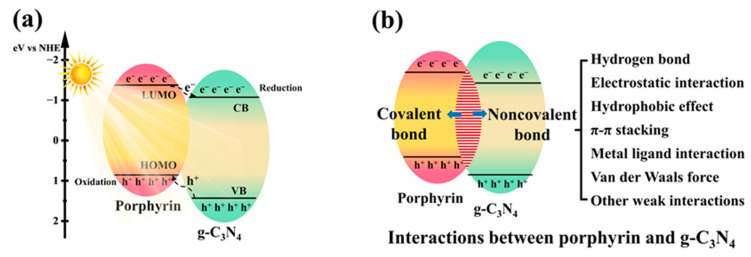
Schematic illustration of the energy band diagram (**a**) and interactions (**b**) between porphyrin and g-C_3_N_4_.

Porphyrins are a class of heterocyclic macromolecules with a rigid skeleton (porphin, depicted in Figure 3a) and a highly π-conjugated system. They are widely found in nature (heme and chlorophyll, as shown in Figure 3b,c) and play a crucial role in essential biological processes such as photosynthesis, oxygen transport, and redox reactions [16,17]. In the photosynthesis process, porphyrins and their derivatives serve as chromophores, absorbing solar energy and converting it into chemical energy. Due to their advantageous features, including excellent redox properties, remarkable visible light harvesting capacity (with extinction coefficient of approximately 10^5^ L·mol^−1^·cm^−1^) [17], high emission intensity, moderate stability, ease of structural modification and functionalization, a wide range of porphyrin-based photocatalysts have been developed, exhibiting captivating optical properties and promising photocatalytic performance [18]. However, as molecular catalysts, porphyrins face certain limitations, such as photo-corrosion under prolonged light irradiation and difficulties in recycling once the reaction is complete. One feasible strategy to address these challenges is to combine g-C_3_N_4_ with porphyrin. This hybrid approach allows for the simultaneous mitigation of the weaknesses associated with g-C_3_N_4_ and porphyrin. Firstly, the exceptional photosensitivity of porphyrin significantly expands the range of visible light absorption. Secondly, the highest occupied molecular orbital (HOMO) and lowest unoccupied molecular orbital (LUMO) positions of porphyrin align well with the band structure of g-C_3_N_4_ (as shown in Figure 4a), thereby facilitating the separation and transmission of charge carriers. Additionally, the strong interface interaction between the two components establishes efficient electron transfer pathways and reduces the migration distance (as depicted in Figure 4b). Lastly, the copolymerization strategy employed to combine porphyrin and g-C_3_N_4_ can optimize the properties of g-C_3_N_4_, such as its specific surface area, as the introduction of porphyrin as a comonomer alters the original polymerization pathway.

**Figure 5:**
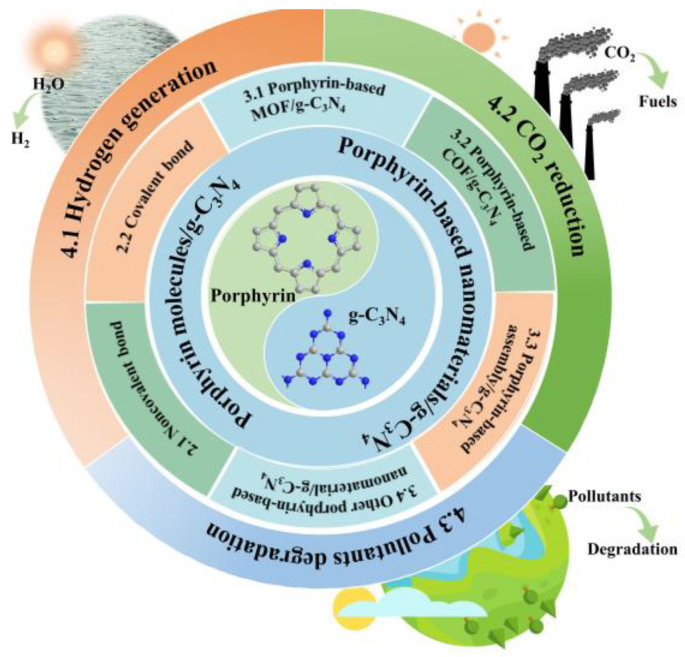
Schematic illustration of the framework and the sections of this review.

The porphyrin/g-C_3_N_4_ hybrid materials are anticipated to demonstrate superior photocatalytic performance compared to their individual components. Significantly, substantial progress has been achieved in recent years. This review (Figure 5) focuses on the fabrication of these composites and their application in solar energy utilization and conversion, which includes photocatalytic hydrogen generation, CO_2_ reduction, and pollutant degradation. Ultimately, conclusions and further prospects on the porphyrin/g-C_3_N_4_ composites are put forward, which would accelerate the deeper understanding and the more extensive application of the porphyrin/g-C_3_N_4_ composites for solar energy utilization.

## 2. Fabrication of Porphyrin Molecules/g-C_3_N_4_ Composite Photocatalysts

A series of porphyrin molecules/g-C_3_N_4_ composites have been prepared to date and have exhibited improved photocatalytic activity compared to pristine g-C_3_N_4_, and the research showed that the properties and the photocatalytic activities are all clearly impacted by the interactions between porphyrin molecules and g-C_3_N_4_.

### 2.1. Noncovalent Bond Interactions between Porphyrin Molecules and g-C_3_N_4_

**Figure 6:**
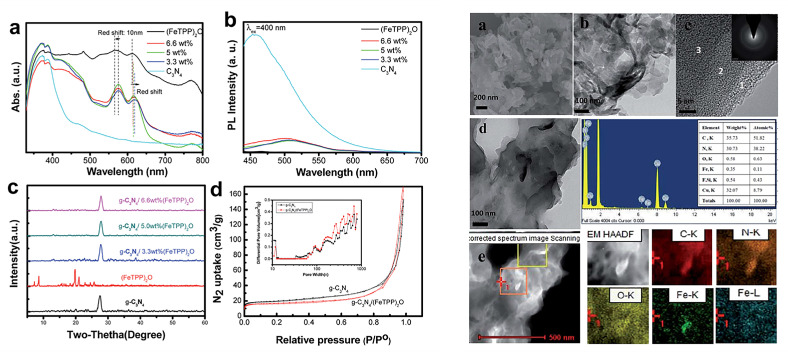
UV-vis diffuse reflection spectra (a), photoluminescence spectra (b), XRD patterns (c), nitrogen adsorption-desorption isotherms (d) of g-C_3_N_4_, (FeTPP)_2_O and g-C_3_N_4_/(FeTPP)_2_O composites (**left**) and SEM (a), TEM (b), HRTEM (c) images and SAED pattern, TEM images (d) and the corresponding EDS (e), mapping images of the g-C_3_N_4_/(FeTPP)_2_O heterostructure (**right**). Reproduced from [19] with permission from [Royal Society of Chemistry].

**Figure 7:**
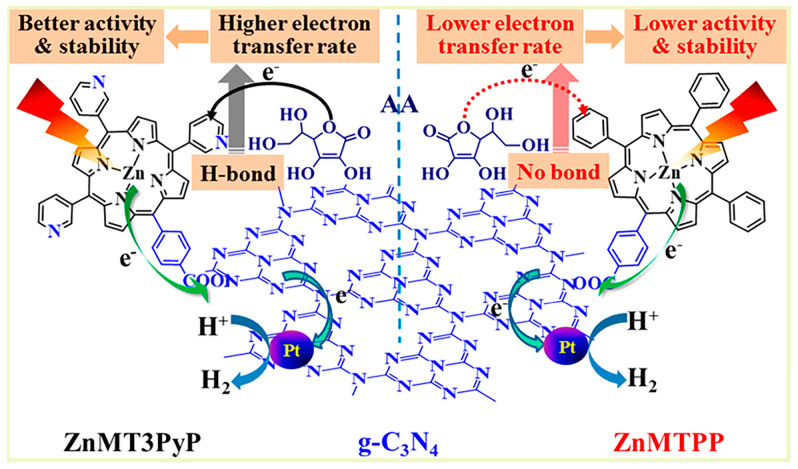
ZnMT_3_PyP and ZnMTPP sensitized graphitic carbon nitride for efficient visible light driven H_2_ production. Reproduced from [20] with permission from [American Chemical Society].

**Figure 8:**
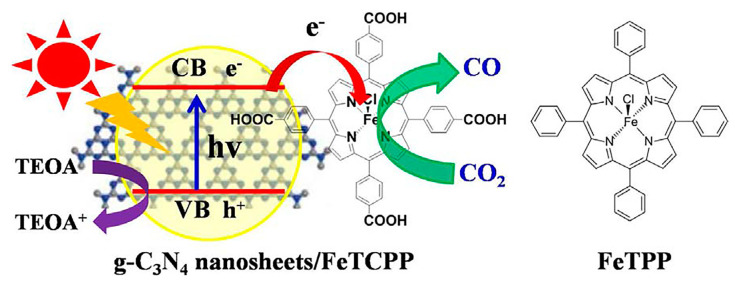
Structure of g-C_3_N_4_/FeTCPP heterogeneous catalyst system and FeTPP. Reproduced from [22] with permission from [Elsevier].

Noncovalent bond interactions mainly include hydrogen bonds, electrostatic interaction, hydrophobic effect, π-π stacking, metal ligand interaction, van der Waals force, and other weak interactions. Porphyrin molecules and g-C_3_N_4_ both possess conjugated structures with abundant surface functional groups, it is easy to compound these two together. The Yao group [19] successfully fabricated a pure organic heterostructure of g-C_3_N_4_/μ-oxo dimeric iron(III) porphyrin [(FeTPP)_2_O] (Figure 6) through π-π stacking and Fe-amine interactions. This was made possible due to the flexible 2D structure and ample amine groups present in g-C_3_N_4_. The experiment demonstrated that the porphyrin (FeTPP)_2_O not only acted as a photosensitizer but also served as a charge promoter, effectively suppressing the recombination of photo-induced electrons and holes in g-C_3_N_4_. Li et al. [20] utilized different asymmetric zinc porphyrin (ZnPy) molecules as photosensitizers, adsorbing them onto the surface of g-C_3_N_4_ (Figure 7). The results revealed that porphyrin molecules with three 3-pyridine substitutions exhibited significantly enhanced photocatalytic activity and stability on Pt/g-C_3_N_4_ compared to those with three phenyl groups. The observed differences were mainly attributed to the rapid electron transfer between porphyrin molecules containing pyridine groups and g-C_3_N_4_. In another study by the Yan group [21], tetra(4-carboxyphenyl) porphyrin (TCPP) was loaded onto the surface of Pt/g-C_3_N_4_ through a simple adsorption process, resulting in the composite TCPP/Pt/g-C_3_N_4_. This organic heterostructure not only improved visible light utilization but also facilitated efficient electron transfer while suppressing the recombination of photo-induced charge carriers. The g-C_3_N_4_/FeTCPP heterogeneous catalyst was prepared by the He group [22] using a straightforward self-assembly approach. In addition to π-π stacking, the interaction between the carboxyl group of FeTCPP and the abundant amino groups of g-C_3_N_4_ formed hydrogen bonds, as illustrated in Figure 8. The resulting g-C_3_N_4_/FeTCPP photocatalysts exhibited remarkable activity in CO_2_ reduction, with a CO evolution rate exceeding 1 mmol g^−1^ h^−1^ and a selectivity of 98%. Zhao et al. [23] conveniently immobilized cobalt meso-tetra-phenylporphyrin (CoTPP) onto g-C_3_N_4_ through π-π supramolecular interaction via self-assembly. The pores induced by the π-π stacking of g-C_3_N_4_ and CoTPP also provided ample space for CO_2_ adsorption. CoTPP demonstrated excellent CO_2_ photoelectrocatalytic activity, characterized by low overpotential and long-term stability. Formic acid was generated as a liquid reduction product, exhibiting a prominent TON value and high selectivity of nearly 100%. To study the effect of carboxyl groups and their peripheral position on H_2_ generation from water splitting [24], free-base porphyrins (TPP, *m*TCPP, *p*TCPP) were used to sensitize g-C_3_N_4_ hybrid photocatalysts through impregnation via noncovalent interactions. All the hybrids displayed enhanced photocatalytic performance for hydrogen evolution compared to pure g-C_3_N_4_. Zhu et al. [25] synthesized a series of photocatalysts by combining g-C_3_N_4_ with metal-porphyrins (MTPPs) through a facile solvothermal method. Notably, CoTPP/g-C_3_N_4_ exhibited excellent activity in the photodegradation of rhodamine B (RhB) when compared to pure g-C_3_N_4_. The enhanced photocatalytic activity was attributed to the more effective interfacial charge transfer between CoTPP and g-C_3_N_4_. The Zhu group [26] synthesized a series of naphthalimide-porphyrin (NP)/g-C_3_N_4_ hybrids, where planar NP molecules were adsorbed onto the 2D g-C_3_N_4_ through π-π stacking. The introduction of NP efficiently inhibited charge recombination and facilitated Förster energy transfer from the donor (g-C_3_N_4_) to the acceptor (NP) due to the overlap between the absorption spectrum of NP and the emission of g-C_3_N_4_. As a result, the photocatalytic hydrogen production performance of 2%NP/g-C_3_N_4_ surpassed that of ZnTCPP/g-C_3_N_4_ and ZnTPP/g-C_3_N_4_. The Abrahamsson group [27] prepared a hybrid assembly of carbon nitride quantum dots (CNQDs) and Fe-porphyrin (Fe-p-TMA), where CNQDs functioned as a visible light absorber and Fe-p-TMA acted as the catalyst for CO_2_ to CO conversion. The interactions between CNQDs and Fe-porphyrin involved electrostatic forces and π-π stacking, establishing a more direct charge transfer pathway that greatly facilitated the CO_2_ to CO transformation.

**Figure 9:**
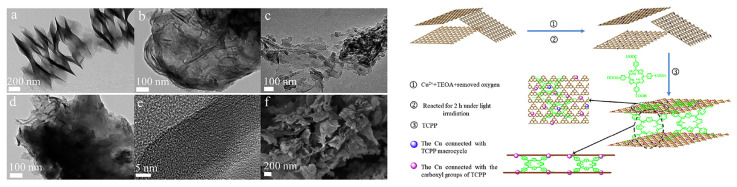
TEM images of TCPP (a), pure g-C_3_N_4_ (b), g-C_3_N_4_-TCPP (c), and g-C_3_N_4_-Cu-TCPP (d), high resolution transmission electron microscopy image (e) and SEM image (f) of the g-C_3_N_4_-Cu-TCPP (**left**), and the formation scheme of the g-C_3_N_4_-Cu-TCPP composite (**right**). Reproduced from [28] with permission from [Elsevier].

**Figure 10:**
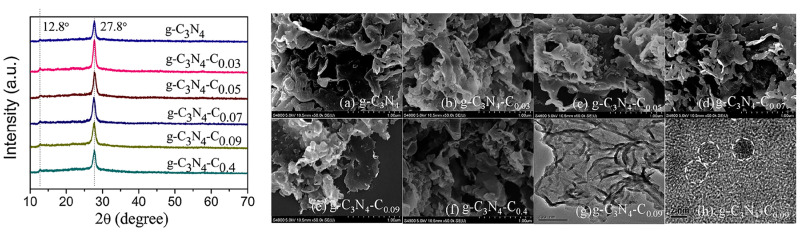
XRD patterns (**left**), SEM, TEM, and HR-TEM images (**right**) of as-synthesized g-C_3_N_4_-C_x_ (x = 0∼0.4) samples. Reproduced from [30] with permission from [Elsevier].

**Figure 11:**
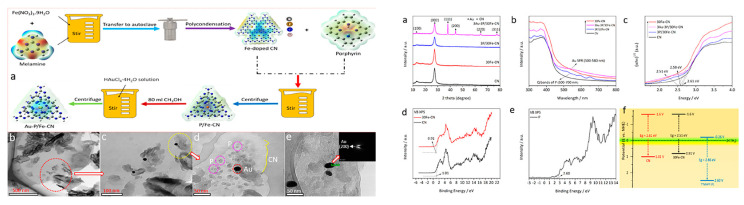
Scheme for the synthesis (a), TEM (b–d), and HR-TEM (e) images of 3Au-3P/30Fe-CN photocatalyst (**left**), XRD patterns (a), absorption spectra (b), and energy band diagram (c–f) of the as-synthesized photocatalyst samples (**right**). Reproduced from [31] with permission from [Elsevier].

In addition to the aforementioned two-component hybrids, several tricomponent composites based on porphyrin/g-C_3_N_4_ have also been synthesized. The Li group [28,29] reported copper-bridged g-C_3_N_4_/porphyrin nanocomposites (Figure 9) for enhanced photocatalytic hydrogen generation. In this composite, porphyrin served as visible light absorption antennas, while g-C_3_N_4_ acted as a substrate. The introduction of copper at the interface of g-C_3_N_4_ and porphyrin not only regulated the morphology and structure of the nanocomposite but also strengthened the interaction between g-C_3_N_4_ and porphyrin. This facilitated effective electron transfer, resulting in a significant enhancement of the photocatalytic activity. The He group [30] synthesized the hybrid photocatalyst g-C_3_N_4_-C0.05/FeTCPP (Figure 10), where g-C_3_N_4_ incorporated with carbon dots acted as the photosensitizer and FeTCPP served as the molecular catalyst. The introduction of trace amounts of carbon dots provided an alternative channel for electron transfer, promoting interfacial charge separation. This resulted in improved photocatalytic performance. Wang et al. [31] fabricated a Z-scheme system, Au-P/Fe-CN (Figure 11), by creating a strong interfacial contact through electrostatic interaction and proper band alignment. The deposition of Au nanoparticles over the surface of P/Fe-CN promoted surface redox properties by enhancing charge carrier separation and transfer efficiency. Multiporphyrin@g-C_3_N_4_ was prepared [32] by binding multi-porphyrin arrays onto the surface of oxidized g-C_3_N_4_, which bears plenty of hydroxyl and carboxyl functional groups. PdCl_4_^2−^ was employed as a connector in this composite. The experimental results demonstrated that the porphyrin molecules not only acted as photosensitizers but also functioned as charge promoters, reducing the recombination of excited electrons and holes in g-C_3_N_4_.

### 2.2. Covalent Bond Interactions between Porphyrin Molecules and g-C_3_N_4_

As we are aware, the efficiency of electron separation and transmission increases with the strengthening of the interaction between porphyrin and g-C_3_N_4_. Considering g-C_3_N_4_ as an organic polymeric semiconductor, it is feasible to establish a covalent bond connection with porphyrin, which would significantly enhance electron transfer. The Ye group [33] was the first to report a hybrid composite of covalently linked Co-porphyrin and low-molecular-weight g-C_3_N_4_. In this composite, Co-porphyrin acted as the reaction center, while g-C_3_N_4_ served as a visible light harvesting antenna. The efficient transmission and accumulation of electrons by the active Co centers, along with the affinity of Co-porphyrin for CO_2_, contributed to the superior photocatalytic performance. This research revolutionized the traditional semiconductor-cocatalyst composite approach.

**Figure 12:**
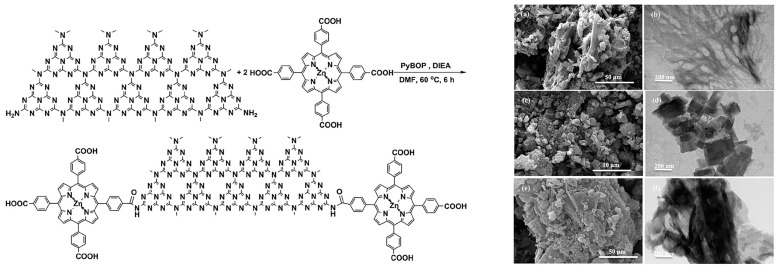
Synthesis of ZnTCPP/g-C_3_N_4_ composites (**left**), and SEM (a,c,e) and TEM (b,d,f) images of g-C_3_N_4_, ZnTCPP, and ZnTCPP/g-C_3_N_4_ (**right**). Reproduced from [34] with permission from [Elsevier].

Post-synthetic modification represents another common approach to covalently link porphyrin with g-C_3_N_4_, taking advantage of the abundant amine groups present at the edges of g-C_3_N_4_ nanosheets. The construction of a ZnTCPP-sensitized g-C_3_N_4_ composite, ZnTCPP/g-C_3_N_4_ (Figure 12), was achieved [34], where ZnTCPP was condensed onto the surface of g-C_3_N_4_ through amide groups serving as bridging units. The significantly enhanced photocatalytic performance of this composite was attributed to the highly efficient separation of electron-hole pairs and the promotion of solar energy utilization, both resulting from the introduction of ZnTCPP via covalent connection.

**Figure 13:**
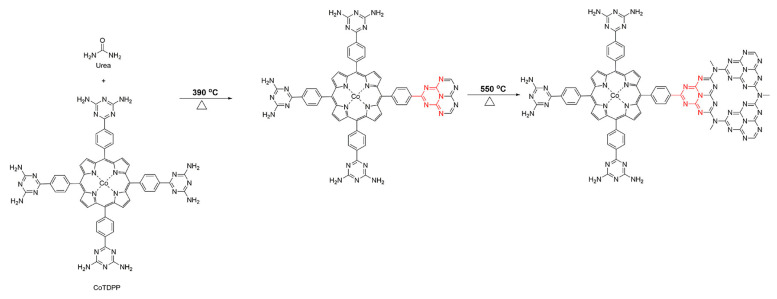
The covalent interaction between CoTDPP and g-C_3_N_4_. Reproduced from [36] with permission from [Springer].

Although covalent interactions between porphyrin and g-C_3_N_4_ can enhance the separation of photo-induced charges through post-synthetic modification, they do not address the limitations of g-C_3_N_4_, such as its limited specific surface area. Copolymerization, however, is a universal strategy for incorporating small organic molecules with suitable functional groups into the g-C_3_N_4_ framework. Building upon this approach, 5,10,15,20-tetrakis(4-(2,4-diamino-1,3,5-triazinyl) phenyl)porphyrin (TDPP) and the corresponding metal porphyrin (MTDPP) were designed and synthesized [35,36] as co-monomers to copolymerize with urea, enabling the construction of covalently connected porphyrin and g-C_3_N_4_ (Figure 13). The resulting photocatalysts, g-CNU-TDPP and g-CNU-MTDPP, not only exhibited an expanded absorption range of visible light but also demonstrated an enlarged specific surface area. Additionally, the separation efficiency of photo-generated electron-hole pairs was significantly improved due to the presence of covalent bonds between TDPP, MTDPP, and g-C_3_N_4_.

## 3. Fabrication of Porphyrin-Based Nanomaterials/g-C_3_N_4_ Composite Photocatalysts

Various nanomaterials based on porphyrin, such as metal-organic frameworks (MOFs), covalent organic frameworks (COFs), and assemblies, have been successfully fabricated [37,38,39,40,41,42,43,44,45,46,47] owing to their abundant functional groups and conjugated rigid planar structure. In addition to porphyrin molecules/g-C_3_N_4_ hybrids, a wide range of porphyrin-based nanomaterials/g-C_3_N_4_ composites have also been developed for solar energy utilization and conversion.

### 3.1. Porphyrin-Based MOF/g-C_3_N_4_ Hybrids for Solar Energy Utilization and Conversion

**Figure 14:**
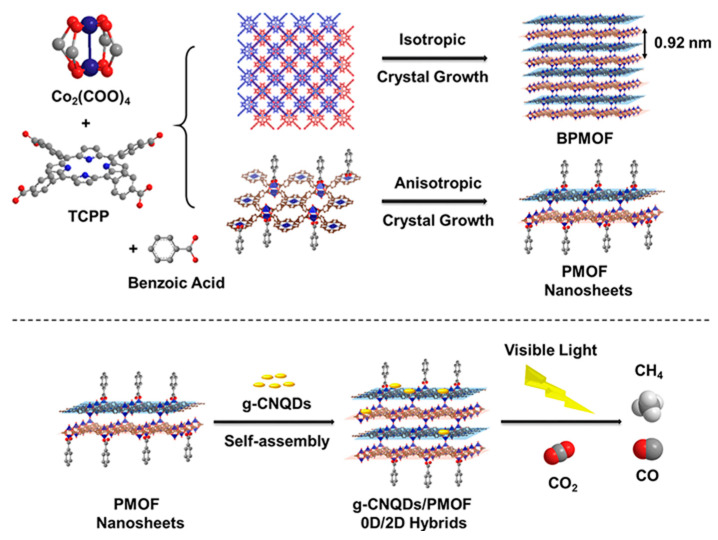
2D PMOF hybrids with g-C_3_N_4_ through coordination interaction. Reproduced from [53] with permission from [American Chemical Society].

**Figure 15:**
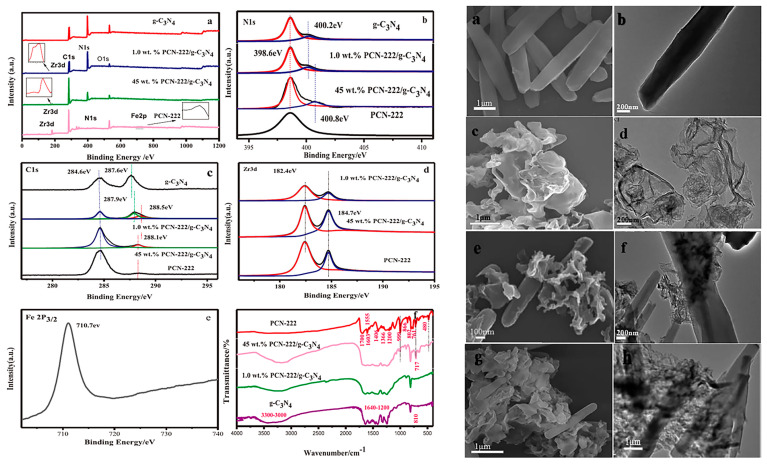
High-resolution XPS (a–d) and FT-IR (e,f) spectra (**left**), and SEM (a,c,e,g) and TEM (b,d,f,h) images of PCN-222, g-C_3_N_4_, and PCN-222/g-C_3_N_4_ (**right**). Reproduced from [55] with permission from [Elsevier].

The unique symmetrical macro-ring structure of porphyrin, combined with its different functional substituents, makes it a prominent ligand for coordinating with metal ions to construct highly stable and porous MOF materials with rich pore structures [48,49,50,51,52]. These MOFs find wide applications in catalysis. Moreover, porphyrin-based MOFs combined with g-C_3_N_4_ for photocatalysis have been extensively studied. A novel hybrid material was prepared [53] by combining zero-dimensional (0D) carbon nitride quantum dots (g-CNQDs) with 2D ultrathin porphyrin through coordination interactions (Figure 14). This hybrid material exhibited shortened migration pathways for photo-induced electrons and gaseous substrates from g-CNQDs to Co active sites. The efficient separation of electron-hole pairs and the long-term capture of electrons at Co centers contributed to the photocatalytic activity and selectivity of the hybrid catalyst. In another study by Zhao group [54], a hybrid photocatalyst was constructed by compounding 2D Cu-porphyrin MOF with g-C_3_N_4_ via π-π stacking. This photocatalyst exhibited outstanding selectivity in CO_2_ reduction under aerobic conditions. The interaction between the 2D-MOF and g-C_3_N_4_ resulted in the formation of a type II heterojunction, where photo-induced holes migrated to the g-C_3_N_4_ moiety while electrons moved to the 2D-MOF unit. Li et al. [55] developed a heterojunction PCN-222/g-C_3_N_4_ (Figure 15) through a facile one-pot solvothermal approach. The hybrid material showed a broadened light response range, enhanced separation efficiency of photo-generated carriers, and improved adsorption capacity for organic pollutants, leading to superior photocatalytic activity. In the work by Shi group [56], a 2D/2D hybrid photocatalyst was fabricated by combining acidified boron-doped g-C_3_N_4_ (HBCNN) with cobalt porphyrin MOF through coordination connections. The interfacial Co-N bond formed a pseudo-gap in the up-spin channel of electronic states, promoting efficient electron-hole separation. The Li group [57] developed a 2D/2D g-C_3_N_4_/PMOF nanohybrid utilizing the interfacial linking of cobalt ions. The presence of cobalt ions with various oxidation states facilitated charge separation and enhanced photocatalytic activity. The sensitization of PMOF resulted in an extended visible light absorption range, while the close and extensive contact at the 2D interface further contributed to the increased photocatalytic performance.

### 3.2. Porphyrin-Based COF/g-C_3_N_4_ Hybrids for Solar Energy Utilization and Conversion

**Figure 16:**
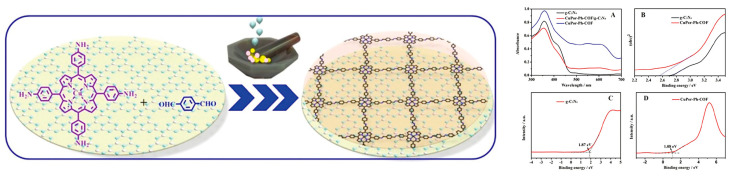
The preparation of CuPor-Ph-COF/g-C_3_N_4_ by the liquid-assisted grinding approach (**left**), and UV-vis diffuse reflection spectra (A), band gap (B–D) of g-C_3_N_4_ and CuPor-Ph-COF/g-C_3_N_4_ (**right**). Reproduced from [58] with permission from [Royal Society of Chemistry].

2D COFs are an enchanting class of materials that possess large π-systems and can be easily functionalized with various substituents. Porphyrin, with its easily modifiable structure, is a natural choice as a monomer for synthesizing COFs. The π-stacking aromatic subunits of porphyrin-based COFs provide a convenient pathway for electronic transport between layers. In Figure 16, a 2D heterojunction of porphyrin COFs/g-C_3_N_4_ was created through the in-situ synthesis of CuPor-Ph-COF on the surface of g-C_3_N_4_ using a benign liquid-assisted grinding approach [58]. This method effectively reduced the recombination of photogenerated electron-hole pairs and facilitated faster separation of photo-induced charges. As a result, the photocatalyst exhibited superior catalytic performance compared to pure g-C_3_N_4_.

### 3.3. Porphyrin-Based Assembly/g-C_3_N_4_ Hybrids for Solar Energy Utilization and Conversion

**Figure 17:**
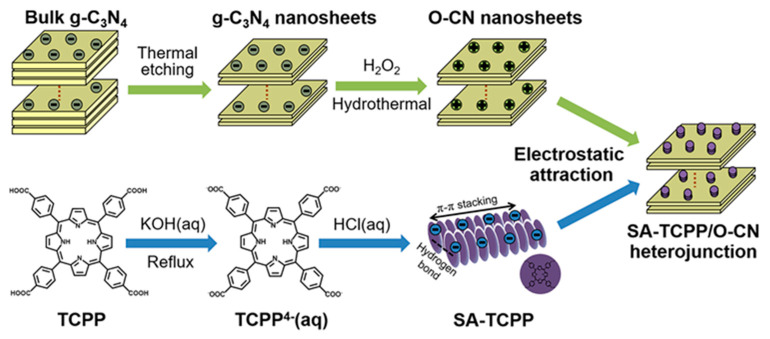
Self-assembly TCPP compound with oxidized g-C_3_N_4_ via electrostatic interaction. Reproduced from [59] with permission from [Elsevier].

**Figure 18:**
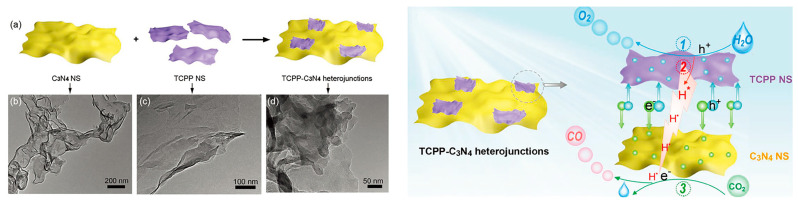
Schematic illustration (a) of the construction of TCPP-C_3_N_4_ heterojunctions and the corresponding TEM (b–d) images (**left**), schematic illustration (of the reaction mechanism on TCPP-C_3_N_4_ heterojunctions (**right**). Reproduced from [60] with permission from [Wiley].

Porphyrin serves as a natural building block for constructing well-defined assemblies through various noncovalent interactions, such as π-π stacking, electrostatic interactions, coordination bonding, hydrogen bonding, and other van der Waals forces. The self-assembly of porphyrin molecules into ordered spatial arrangements not only expands the absorption range of visible light but also enhances photo-induced electron transmission and improves photostability under long-term irradiation. In a study by the Zhu group [59], an all-organic, self-assembled heterojunction photocatalyst, consisting of TCPP (SA-TCCP) and oxidized g-C_3_N_4_ (O-CN), was synthesized using an in situ electrostatic approach (Figure 17). The light-harvesting capacity of O-CN was greatly enhanced by the broad-spectrum responsive ability of SA-TCCP. The π-π interaction between SA-TCCP and O-CN facilitated electron delocalization, while the well-matched band structures generated a built-in electric field, promoting interfacial charge transfer. The synergistic effects between SA-TCCP and O-CN contributed to the enhanced photocatalytic oxidation activities. Kuang et al. [60] designed a heterojunction C_3_N_4_-supramolecular TCPP nanosheet (NS) (Figure 18) with efficient charge separation based on van der Waals forces. The porphyrin NS acted as a water oxidation booster, while the C_3_N_4_ NS synergistically functioned as the CO_2_ reduction center. The TCPP-C_3_N_4_ heterojunction regulated charge migration properties and accelerated robust water oxidation kinetics, thereby enhancing the photocatalytic CO_2_ reduction activity. Furthermore, a TCPP nanofibers/g-C_3_N_4_ nanocomposite was fabricated [61] using a self-assembly approach. The charge separation efficiency was improved through an enhanced exciton-coupled charge transfer process between TCPP aggregates and g-C_3_N_4_. Additionally, the optimized capture capacity of photon energy contributed to the promoted photodegradation efficiency.

### 3.4. Other Porphyrin-Based Nanomaterial/g-C_3_N_4_ Hybrids for Solar Energy Utilization and Conversion

**Figure 19:**
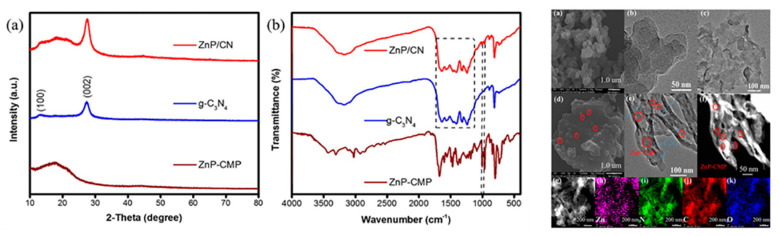
XRD patterns (a) and FT-IR (b) spectra of ZnP/CN, g-C_3_N_4_, and ZnP-CMP (**left**), SEM (a,d), TEM (b,c,e,f), and element mapping (e–k) images of ZnP/CN, g-C_3_N_4_, and ZnP-CMP (**right**). Reproduced from [62] with permission from [Elsevier].

Except for the aforementioned porphyrin/g-C_3_N_4_ hybrids, there are additional nanomaterials based on porphyrin coupled with g-C_3_N_4_ that are utilized for solar energy applications. In a study conducted by the Ji group [62], a zinc porphyrin polymer/g-C_3_N_4_ heterojunction (Figure 19) was reported for photocatalytic synthesis. The hybrid photocatalyst performance was enhanced by the improved visible-light harvesting ability and photo-induced charge mobility resulting from the incorporation of zinc-porphyrin-conjugated microporous polymer. Another photocatalyst, a porphyrin-based metal-organic cages (MOC)/g-C_3_N_4_ heterostructure, was prepared [63] through π-π stacking to facilitate photocatalytic hydrogen generation in a direct Z-scheme configuration. The direct Z-scheme heterostructure promoted charge transmission, expanded the light absorption range, protected the cages from degradation within the hybrid, and ultimately enhanced the photocatalytic activity of the system.

## 4. Photocatalytic Applications of Porphyrin/g-C_3_N_4_ Composite for Solar Energy Utilization and Conversion

Due to their potential applications in solar energy utilization and conversion, semiconductor-based photocatalysts have garnered significant attention in the field of energy and the environment. Photocatalytic reactions typically involve three steps [64,65,66]: (1) Generation of charge carriers: when semiconductors absorb light with energy equal to or greater than their corresponding bandgaps, photocarriers are generated. Excited by photons, electrons (e^−^) move from the valence band (VB) to the conduction band (CB), creating positively charged holes (h^+^) in the VB. The redox abilities of electrons and holes are determined by the positions of the CB and VB. (2) Migration of charge carriers: within the semiconductor, some charge carriers recombine due to defects, collisions, and other factors, while others migrate to the surface of the semiconductor. (3) Involvement of charge carriers in reactions: active electrons or holes on the surface of the semiconductor, either with or without the assistance of co-catalysts, participate in photoreduction or photooxidation reactions with absorbed reactants. Porphyrin/g-C_3_N_4_ composites have been extensively studied for solar energy utilization and conversion, including photocatalytic hydrogen production, CO_2_ reduction, and pollutant degradation. These composites exhibit improved visible light harvesting ability, enhanced separation efficiency of charge carriers, and long-term stability. In this section, we briefly review the applications of porphyrin/g-C_3_N_4_ composites in the aforementioned areas.

### 4.1. Photocatalytic Water Splitting for Hydrogen Generation

Hydrogen is a promising green energy with the highest energy density (140 MJ kg^−1^), which is dramatically higher than that of traditional hydrocarbon fuels (40-50 MJ kg^−1^) [67]. Thus, the generation of hydrogen from water splitting utilizing semiconductor and solar energy is an appealing and promising substitute for fossil fuel [68]. Water splitting involves generation of oxygen as one product and formation of hydrogen as the other (Equations (1) and (2)). The overall transformation (Equation (3)) is a multielectron route advanced by solar energy and semiconductors, the bandgap of the semiconductor should be at 1.23~3.23 eV and the CB potential should be higher than hydrogen electrode potential.

Oxidation: H_2_O + 2h^+^ → 2H^+^ + 1/2O_2_ (1)

Reduction: 2H^+^ + 2e^−^ → H_2_ (2)

Overall reaction: H_2_O → H_2_ + 1/2O_2_ (3)

The photocatalysts comprising porphyrin/g-C_3_N_4_ composites enhance charge transfer, thereby accelerating the rate of hydrogen evolution. This section primarily focuses on the research related to hydrogen evolution utilizing porphyrin/g-C_3_N_4_ composites. Table 1 provides a summary of the advancements in hydrogen generation through water splitting using porphyrin/g-C_3_N_4_ composites.

**Figure 20:**
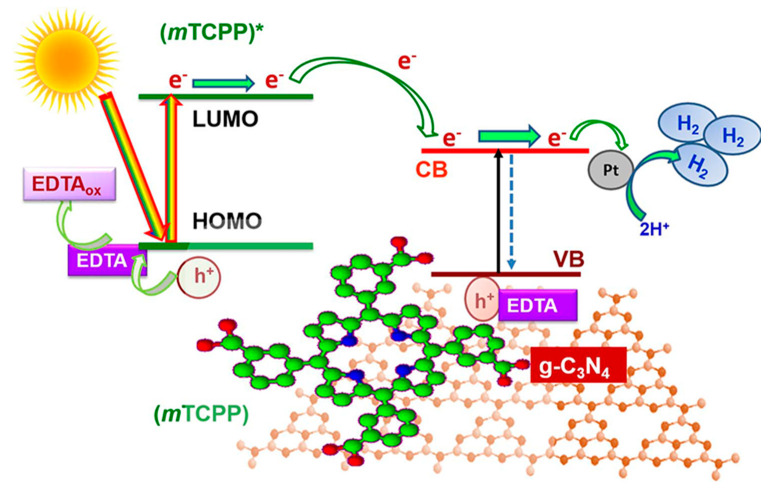
Proposed mechanism for photocatalytic hydrogen production from water over g-C_3_N_4_ sensitized with *m*TCPP, the (*m*TCPP) * means the excited state of *m*TCPP. Reproduced from [24] with permission from [Elsevier].

Oxidizing water to produce oxygen is a challenging process, and current research primarily focuses on the half reaction of hydrogen evolution (Equations (1)–(3)). Alongside the photocatalyst and solar energy, sacrificial agents play a crucial role in harvesting unreacted holes to facilitate proton reduction, while co-catalysts are necessary under specific reaction conditions (Figure 20). Table 1 demonstrates that among the sacrificial agents, triethanolamine (TEOA) has proven to be the most efficient and widely employed for hydrogen generation. Additionally, when porphyrin molecules are combined with g-C_3_N_4_ photocatalysts, the assistance of co-catalyst Pt is generally required to achieve excellent hydrogen evolution activity. In the case of porphyrin/g-C_3_N_4_ Z-scheme systems, the presence of Pt as a co-catalyst is typically unnecessary due to their unique electron-hole transport mode and the higher reduction ability of their corresponding CB. As depicted in Table 1, the photocatalytic activities are commonly evaluated by measuring the hydrogen evolution rate, which often reaches up to mmol per gram of porphyrin/g-C_3_N_4_ composites within one hour. To account for variations in experimental instruments and other conditions, the apparent quantum efficiency (AQY) is also employed to assess the performance of photocatalysts and measure the efficiency of solar energy utilization.

### 4.2. Photocatalytic CO_2_ Reduction

CO_2_ is an integral part of the atmosphere and plays a crucial role in the global carbon cycle. The continuously increasing concentration of CO_2_, resulting from the explosive growth of the world’s population and economic activities, has led to global warming and ecological changes due to its status as a leading greenhouse gas. Global energy demand, including fossil fuels, which are the main sources of CO_2_ emissions, will continue to grow for a certain period. Inspired by natural photosynthesis, the photocatalytic reduction of CO_2_ to reduce fuels such as methane, carbon monoxide, methanol, and other valuable chemicals has been recognized as a promising approach to address energy and environmental issues. This approach achieves two goals simultaneously [69,70]. However, the linear molecular geometry and the high bond energy of C=O present certain challenges to CO_2_ reduction, both from thermodynamic and dynamic perspectives. Similar to photocatalytic water splitting, photocatalytic CO_2_ reduction also involves both the CO_2_ reductive half-reaction and the water oxidative half-reaction in order to achieve carbon neutrality. The single-electron reduction of CO_2_ requires extremely high energy, as shown in Equation (4). Therefore, the half-reaction typically follows a proton-assisted multi-electron pathway. The process involves the gradual acquisition of multiple electrons and hydrogen radicals to produce gaseous and liquid products such as CH_4_, CO, HCHO, and CH_3_OH, as presented in Equations (5)–(9) [71].

CO_2_ + e^−^ → CO_2_^−^ (E^o^ = −1.90 V vs. NHE at pH 7) (4)

CO_2_ + 2H^+^ + 2e^−^ → HCOOH (E^o^ = −0.61 V vs. NHE at pH 7) (5)

CO_2_ + 2H^+^ + 2e^−^ → CO + H_2_O (E^o^ = −0.53 V vs. NHE at pH 7) (6)

CO_2_ + 4H^+^ + 4e^−^ → HCHO + H_2_O (E^o^ = −0.48 V vs. NHE at pH 7) (7)

CO_2_ + 6H^+^ + 6e^−^ → CH_3_OH + H_2_O (E^o^ = −0.38 V vs. NHE at pH 7) (8)

CO_2_ + 8H^+^ + 8e^−^ → CH_4_ + H_2_O (E^o^ = −0.24 V vs. NHE at pH 7) (9)

The selectivity of the reduced products is primarily influenced by the structures of the photocatalyst. Hybrid heterojunctions between porphyrin and g-C_3_N_4_ have been extensively utilized for CO_2_ reduction. The photocatalytic performance, including activity and selectivity, is summarized in Table 2.

**Figure 21:**
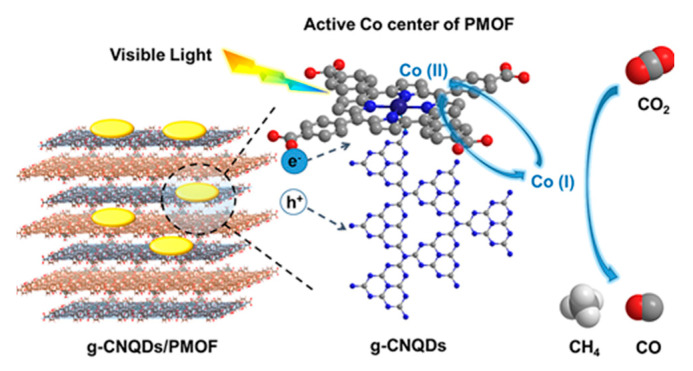
Proposed mechanism of CO_2_ reduction over g-CNQDs/PMOF hybrids under visible light irradiation. Reproduced from [53] with permission from [American Chemical Society].

As can be seen, the photocatalytic reduction of CO_2_ primarily occurs in either the liquid or gas phase. In the liquid phase reaction, the photocatalyst is uniformly dispersed in a CO_2_-saturated solution. In the gas-phase reaction system, on the other hand, the photocatalyst is typically fixed on a substrate support, and a mixture of CO_2_ and water vapor directly reacts with the photocatalyst. In the liquid phase reaction, sacrificial agents such as TEOA and triethylamine (TEA) are essential in the CO_2_ reductive half-reactions to consume excessive generated holes, similar to the half-reaction in photocatalytic water splitting. Acetonitrile (CH_3_CN) is often chosen as the solvent in the reaction system for two reasons: (1) Organic solvents have higher solubility for CO_2_ gas compared to water, and (2) Competitive hydrogen evolution reactions can be effectively inhibited. Additionally, a certain amount of water is necessary as it can supply protons for participation in the proton-coupled multi-electron reduction of CO_2_. The gas-phase reaction is independent of the solvent and sacrificial agent and represents a relatively simple reaction system. In porphyrin/g-C_3_N_4_ hybrid photocatalysts, g-C_3_N_4_ typically acts as a photosensitizer to absorb visible light and generate electrons, while porphyrin, particularly metal porphyrin, with metal ions (such as Fe, Co) at the center of the porphyrin ring, serves as the catalytic active site for reducing the adsorbed CO_2_ to reduced products (Figure 21). However, there are exceptions, such as in the case of Cu-Zn-TCPP/g-C_3_N_4_ photocatalyst, where the Cu node in the 2D-MOF moiety is hydroxylated and functions as the catalytic center instead of the Zn ion in the porphyrin ring. In another case, the 2D-2D heterojunction TCPP nanosheets/C_3_N_4_ nanosheets catalyzes the overall CO_2_ reduction without the addition of sacrificial agents, with the porphyrin TCPP serving as a water oxidation booster and the C_3_N_4_ nanosheets acting as the CO_2_ reduction center. Regarding the selectivity of products, CO is the main reduction product when employing porphyrin/g-C_3_N_4_ nanocomposites as photocatalysts. In summary, current research based on porphyrin/g-C_3_N_4_ nanocomposites for CO_2_ reduction is still in its initial stages, requiring further progress in fabricating more efficient photocatalysts and understanding the mechanism of CO_2_ reduction.

### 4.3. Photocatalytic Degradation of Pollutants

With the global process of industrialization and the increasing global population, a significant amount of pollutants has been generated and discharged into the environment. This has raised concerns worldwide about sustainability because these pollutants not only contaminate the environment but also pose a threat to human health and well-being. In order to protect our environment and ensure the sustainable development of humanity, photocatalytic degradation of pollutants has emerged as an effective approach to harness solar energy and achieve these goals.

Porphyrin/g-C_3_N_4_ hybrid photocatalysts possess the ability to efficiently absorb solar energy and facilitate rapid charge carrier transfer [72,73]. Consequently, they can be utilized to enhance the performance of photocatalytic pollutant degradation. The recent advancements in this field are summarized in Table 3.

**Figure 22:**
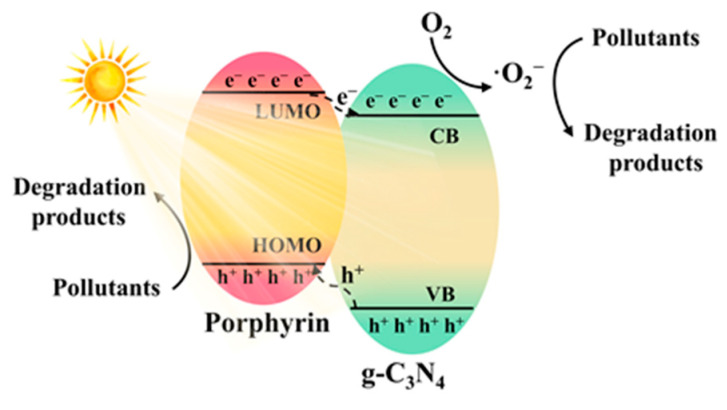
Proposed carrier separation, transfer route of porphyrin/g-C_3_N_4_ composite, and photocatalytic pollution degradation process.

Figure 22 provides a schematic illustration of the photocatalytic degradation of pollutants based on porphyrin/g-C_3_N_4_. Under light irradiation, photo-induced electrons typically migrate from the LUMO of porphyrin to the CB of g-C_3_N_4_ due to the more negative potential of the LUMO orbit of porphyrin. Conversely, holes preferentially transfer from the VB of g-C_3_N_4_ to the HOMO orbit of porphyrin due to the more positive position of the CB of g-C_3_N_4_. The photo-induced electrons tend to react with oxygen molecules absorbed on the g-C_3_N_4_ surface, generating superoxide anion radicals (·O^2−^), which contribute to the degradation of pollutants. Meanwhile, the generated holes tend to react with absorbed OH^−^ groups on the surface of porphyrin, producing hydroxyl radicals (·OH) that facilitate pollutants degradation. As shown in Table 3, the catalytic performance of the photocatalysts was commonly evaluated using pollutants such as RhB, tetracycline (TC), and bisphenol A (BPA). The photocatalytic activities were typically assessed based on the decomposition rate of pollutants within a specified time or the apparent rate constant. The porphyrin/g-C_3_N_4_ composite photocatalysts consistently exhibited significantly improved photocatalytic degradation performance compared to pristine g-C_3_N_4_. This enhancement can be attributed to the constructed heterojunctions, which not only enhance the separation and transportation of photo-generated charge carriers but also contribute to more efficient utilization of solar energy.

## 5. Challenges and Perspectives

Solar energy offers a distinct and natural advantage as a sustainable energy source. It is a renewable and abundant energy that endures over time and is widely accessible. Additionally, it is a completely clean, safe, and reliable energy source, not constrained by mining, transportation, or storage limitations. The most promising approach for solar energy conversion and storage involves chemical bonds. The development of new materials is necessary to harness and convert solar energy effectively. Porphyrin/g-C_3_N_4_ composite photocatalysts have been designed and utilized for capturing solar energy across a broader spectrum and facilitating the separation of photo-generated charges. These catalysts have been successfully employed in photocatalytic hydrogen evolution, CO_2_ reduction, and pollutant degradation. Despite notable progress, research in this field is still in its early stages, and several challenges remain to be addressed.

(1) Although the porphyrin/g-C_3_N_4_ composites broaden the absorption range of visible light, there is a need to further increase their utilization of higher-wavelength solar energy (λ > 500 nm).
(2) The efficiency of separating photo-induced charges should be substantially enhanced as the current efficiency of the porphyrin/g-C_3_N_4_ hybrid falls short of practical application requirements.
(3) Fabrication of uniform single-layer or few-layer porphyrin/g-C_3_N_4_ photocatalysts is necessary to achieve more efficient solar-to-chemical conversion and energy storage.
(4) Addressing the challenge of oxygen evolution is crucial for both water splitting and CO_2_ reduction. The scarcity of cocatalysts with high activity for oxygen evolution necessitates additional efforts.
(5) The mechanism, cocatalysts, reaction pathways, and product selectivity involved in CO_2_ reduction catalyzed by porphyrin/g-C_3_N_4_ photocatalysts remain unclear and require further exploration and research.
(6) The degradation of pollutants using porphyrin/g-C_3_N_4_ hybrid photocatalysts is still in its preliminary stages, and the photocatalytic degradation of gaseous pollutants has received less attention compared to organic pollutants in water.
(7) The stability and recyclability of porphyrin/g-C_3_N_4_ photocatalysts are essential to meet industrial requirements and should be optimized for practical applications in the future.

Looking ahead to the future, porphyrin/g-C_3_N_4_ nanohybrids hold immense potential for applications in the field of artificial photosynthesis. This review aims to provide valuable insights and assistance for future research, fostering significant advancements in solar energy conversion. Progress in the design and synthesis of porphyrin/g-C_3_N_4_ materials will contribute to addressing the energy and environmental challenges we face. Collaborative efforts are essential to propel the practical utilization of porphyrin/g-C_3_N_4_ nanostructures, catalyzing a transformation in renewable energy. The realization of clean energy and environmental goals, achieved through the efficient production of carbon-neutral fuels via artificial photocatalysis, is no longer a distant dream but an achievable reality.

## Figures and Tables

**Table 1:** Summary of porphyrin/g-C_3_N_4_ composite photocatalysts for hydrogen evolution from water.

Composite	Cocatalyst	Sacrificial Agent	Light Source	Activity	AQY	Ref.
C_3_N_4_/[(FeTPP)_2_O]	N/A	10 vol% TEOA	300 W Xe Lamp	187.5 μmol g^−1^ h^−1^	0.0415% (420 nm)	[19]
ZnMT3PyP-Pt/C_3_N_4_	Pt	50 mM AA	300 W Xe Lamp	2.28 mmol g^−1^ h^−1^	25.1% (420 nm)	[20]
TCPP/Pt/g-C_3_N_4_	Pt	10 vol% TEOA	450 W Hg Lamp (λ > 380 nm)	1.21 mmol g^−1^ h^−1^	N/A	[21]
*m*TCPP/CN	Pt	20 mM EDTA	Mercury vapour lamp	2.28 mmol g^−1^ h^−1^	N/A	[24]
NP/g-C_3_N_4_	Pt	10 vol% TEOA	300 W Xe Lamp	2.29 mmol g^−1^ h^−1^	N/A	[26]
g-C_3_N_4_-Cu-TCPP	N/A	10 vol% TEOA	300 W Xe Lamp	1.67 mmol g^−1^ h^−1^	N/A	[28]
g-C_3_N_4_-Cu-THPP	N/A	10 vol% TEOA	300 W Xe Lamp	7.5 μmol h^−1^	N/A	[29]
Au-P/Fe-CN	N/A	20 vol% methanol	300 W Xe Lamp	3.17 mmol g^−1^ h^−1^	3.26% (420 nm)	[31]
g-CNU-TDPP	Pt	10 vol% TEOA	300 W Xe Lamp	7.6 mmol g^−1^ h^−1^	13.3% (450 nm)	[35]
HBCNN/CoPMOF	Pt	AA	225 W Xe Lamp	33.5 mmol g^−1^ h^−1^	N/A	[56]
C_3_N_4_/Co/PMOF	N/A	17 vol% TEOA	300 W Xe Lamp	1.81 mmol g^−1^ h^−1^	N/A	[57]
MOC-Py-Zn/g-C_3_N_4_	N/A	10 vol% TEOA	300 W Xe Lamp	10.3 mmol g^−1^ h^−1^	N/A	[63]
ZnPy-4-Pt/C_3_N_4_	Pt	50 mM AA	300 W Xe Lamp	3.49 mmol g^−1^ h^−1^	32.3% (420 nm)	[68]

**Table 2:** Summary of porphyrin/g-C_3_N_4_ composite photocatalysts for photocatalytic CO_2_ reduction.

Composite	Reaction Solvent	Sacrificial Agent	Light Source	Activity	Ref.
g-C_3_N_4_/0.75% FeTCPP	CH_3_CN:H_2_O = 3:1	20 vol% TEOA	300 W Xe Lamp	CO: 1.09 mmol g^−1^ h^−1^	[22]
CNQD·[Fe-*p*-TMA]	H_2_O	TEOA	Hg-Xe Lamp (55 mW cm^−2^)	CO: 279 μmol h^−1^ TON: >10^5^ Selectivity: 96%	[27]
g-C_3_N_4_-CDs/FeTCPP	CH_3_CN:H_2_O = 3:1	20 vol% TEOA	300 W Xe Lamp	CO: 11.85 mmol g^−1^ h^−1^	[30]
Co-porphyrin/g-C_3_N_4_	CH_3_CN	20 vol% TEOA	300 W Xe Lamp	CO: 17 μmol g^−1^ h^−1^ CH_4_: 0.7 μmol g^−1^ h^−1^ Selectivity: 80%	[33]
g-CNU-CoTDPP	CH_3_CN	20 vol% TEOA	300 W Xe Lamp	CO: 57 μmol g^−1^ h^−1^ H_2_: 14.2 μmol g^−1^ h^−1^ Selectivity: 79%	[36]
g-CNQDs/PMOF	CH_3_CN:H_2_O = 3:1	20 vol% TEOA	300 W Hg Lamp	CO: 16.1 μmol g^−1^ h^−1^ CH_4_: 6.86 μmol g^−1^ h^−1^ Selectivity: 70%	[53]
Cu-Zn-TCPP/g-C_3_N_4_	N/A	TEA	300 W Xe Lamp	CO: 43.75 μmol g^−1^ h^−1^ CH_4_: 323.75 μmol g^−1^ h^−1^ CH_4_ selectivity: 88%	[54]
3% TCPP-C_3_N_4_	N/A	H_2_O	300 W Xe Lamp	CO: 16.8 μmol g^−1^ h^−1^ O_2_: 10 μmol g^−1^ h^−1^	[60]

**Table 3:** Summary of porphyrin/g-C_3_N_4_ composite photocatalysts for photocatalytic pollutant degradation.

Composite	Light Source	Decomposition Rate	Initial Concentration	Ref.
CoTPP/g-C_3_N_4_	350 W Xe Lamp	RhB: 99.79% in 90 min	25 μmol L^−1^	[25]
O-C_3_N_4_@(Pd-TPyP)_3_	320W Xe Lamp	RhB: about 90% in 40 min	10 mg L^−1^	[32]
ZnTCPP/g-C_3_N_4_	300 W Xe Lamp	RhB: 96% in 30 min TC: 80.3% in 120 min	RhB: 10 mg L^−1^ TC: 30 mg L^−1^	[34]
Co-porphyrin/g-C_3_N_4_	300 W Xe Lamp	RhB: 97.9% in 120 min Ofloxacin: 95.9% in 200 min	20 mg L^−1^	[55]
CuPor-Ph-COF/g-C_3_N_4_	300 W Xe Lamp	RhB: 86% in 90 min	10 mg L^−1^	[58]
SA-TCPP/O-CN	500 W Xe Lamp	BPA: 0.150 h^−1^	10 ppm	[59]
C_3_N_4_/TCPP	350 W Xe Lamp	RhB: 0.033 min^−1^	10 ppm	[61]
C_3_N_4_-TCPP/CP	300 W Xe Lamp	RhB: 95% in 150 min	50 mg L^−1^	[72]
0.1% TCPP/g-C_3_N_4_	Xe Lamp	RhB: 0.033 min^−1^	22 ppm	[73]
